# Systemic Mastocytosis Presenting as Non-cirrhotic Portal Hypertension With Upper Gastrointestinal Bleeding in a Young Female Patient: A Case Report

**DOI:** 10.7759/cureus.109267

**Published:** 2026-05-20

**Authors:** Ashish Jangid, Prashant Pandey, Mohammad Shafat I Siddiqui, O.P. Tiwari, Santosh K Pandey, Neeraj K Singh

**Affiliations:** 1 General Medicine, Heritage Institute of Medical Sciences, Varanasi, IND; 2 Internal Medicine, Heritage Institute of Medical Sciences, Varanasi, IND; 3 Pathology, Heritage Institute of Medical Sciences, Varanasi, IND

**Keywords:** esophageal varices, non-cirrhotic portal hypertension, pancytopenia, splenomegaly, systemic mastocytosis, upper gastrointestinal bleeding

## Abstract

Systemic mastocytosis is a rare clonal disease that leads to the abnormal growth and accumulation of mast cells in different organs. Clinical presentation varies from indolent disease to aggressive multiorgan involvement. Portal hypertension due to hepatic infiltration is not common, and this presentation with upper gastrointestinal bleeding is extremely rare, especially in young patients.

We present a case report of a 21-year-old female with melena, generalized weakness, and dizziness. On examination, massive splenomegaly was noted. Laboratory tests revealed pancytopenia, and ultrasonography and contrast-enhanced CT of the abdomen revealed features of portal hypertension without cirrhosis. Upper gastrointestinal endoscopy showed esophageal varices. Systemic mastocytosis was diagnosed by bone marrow examination, which revealed dense aggregates of abnormal spindle-shaped mast cells, followed by immunohistochemistry of the samples, which were positive for CD117 and CD25.

Management of the patient included supportive care such as blood transfusions and endoscopic therapy for variceal bleeding. This case demonstrates a very rare and severe manifestation of systemic mastocytosis in a young patient, and highlights the importance of considering infiltrative hematological disorders in non-cirrhotic portal hypertension presenting with unexplained cytopenias.

## Introduction

Systemic mastocytosis is a rare clonal disease that is characterized by abnormal proliferation and accumulation of mast cells in different organs, most commonly the bone marrow. It is uncommon but a significant diagnostic consideration, estimated at about 1 per 10,000-20,000 people with unexplained systemic manifestations [1,2]. The disease is generally seen in adults, with a median age of diagnosis in the fifth to sixth decade; hence, it is quite unique to present in a young person.

The pathogenesis of the disease mainly consists of activating mutations of the proto-oncogene KIT; the most common mutation is the D816V mutation, which leads to uncontrolled proliferation and accumulation of mast cells [1,3]. Such abnormal mast cells may infiltrate several organs, such as the bone marrow, liver, spleen, and gastrointestinal tract [2,3]. Clinical manifestations are diverse, ranging from indolent disease with minimal symptoms to aggressive disease with severe organ dysfunction.

Although hepatic involvement in systemic mastocytosis is not very common, it is clinically important because hepatic sinusoidal infiltration by mast cells can cause hepatic fibrosis and increased intrahepatic resistance, resulting in non-cirrhotic portal hypertension [4-6]. Although splenomegaly may be seen in advanced disease, portal hypertension and upper gastrointestinal bleeding due to varices are extremely rare presentations [6-8]. Moreover, systemic mastocytosis in young people is generally rare; therefore, such an aggressive manifestation is quite exceptional [2].

We describe a case of a 21-year-old female who presented with massive splenomegaly, non-cirrhotic portal hypertension, and upper gastrointestinal bleeding, who was later diagnosed with systemic mastocytosis on bone marrow biopsy. This case demonstrates a clinical presentation of an infiltrative hematological disorder that is atypical and aggressive in a young patient and highlights the need to consider infiltrative hematological disorders in patients with no known etiology leading to portal hypertension and cytopenias.

## Case presentation

A 21-year-old female with a history of hypothyroidism presented with melena for six days, associated with generalized weakness and dizziness for one day. There was no history of flushing, pruritus, abdominal pain, alcohol consumption, hepatitis, or drug abuse.

On physical examination, the patient was hemodynamically stable, with a pulse rate of 96 beats per minute and a blood pressure of 104/78 mmHg. Pallor was present. Abdominal examination revealed massive splenomegaly extending approximately 25 cm below the left costal margin. Mild hepatomegaly was also noted. There was no evidence of ascites, jaundice, spider angiomas, palmar erythema, or other stigmata of chronic liver disease.

Laboratory investigations showed marked pancytopenia, including severe anemia, leukopenia, and thrombocytopenia. Liver function tests indicated hyperbilirubinemia and minimally elevated alkaline phosphatase, along with a disproportionately elevated alkaline phosphatase level, suggestive of an infiltrative or cholestatic pattern rather than primary hepatocellular injury. Renal function parameters were within normal limits. Coagulation studies revealed mild prolongation of prothrombin time and INR. Tryptase was markedly elevated at 66 ng/mL, supporting mast cell proliferation. Serological testing for hepatitis B virus, hepatitis C virus, and HIV was non-reactive, thereby excluding common infectious causes of chronic liver disease.

Baseline hematological parameters are summarized in Table 1, and liver function test parameters are shown in Table 2.

**Table 1:** Hematological parameters (complete blood count). Abbreviations: RBC: Red Blood Cells; MCV: Mean Corpuscular Volume; RDW: Red Cell Distribution Width

Parameter	Value	Reference Range
Hemoglobin	7.1 g/dL	11 - 16 g/dL
Total Leukocyte Count	1.3 × 10³/mm³	4.5-11 × 10³/mm³
RBC Count	2.4 million/mm³	4.5 - 5.5 million/mm³
Platelet Count	12 × 10³/mm³	150 - 400 × 10³/mm³
MCV	101 fL	80 - 98 fL
Neutrophils	60%	40 - 80%
Lymphocytes	26%	20 - 40%

**Table 2:** Liver function test parameters. Abbreviations: AST: Aspartate Aminotransferase; ALT: Alanine Aminotransferase; ALP: Alkaline Phosphatase; SGOT: Serum Glutamic-Oxaloacetic Transaminase; SGPT: Serum Glutamic-Pyruvic Transaminase

Parameter	Value	Reference Range
Total Bilirubin	1.3 mg/dL	0.3 - 1.2 mg/dL
Direct Bilirubin	0.6 mg/dL	0 - 0.2 mg/dL
SGOT (AST)	48 U/L	10 - 40 U/L
SGPT (ALT)	38 U/L	7 - 56 U/L
Alkaline Phosphatase (ALP)	236 U/L	44 - 147 U/L
Albumin	3.9 g/dL	3.5 - 5.0 g/dL
Total Protein	7.8 g/dL	6.6 - 8.7 g/dL
A:G Ratio	1.0	1.2 - 2.0

Ultrasonography of the abdomen demonstrated marked splenomegaly (Figure 1). Contrast-enhanced CT of the abdomen further confirmed massive splenomegaly, measuring 27.5 cm, with features of portal hypertension (Figure 2).

**Figure 1:**
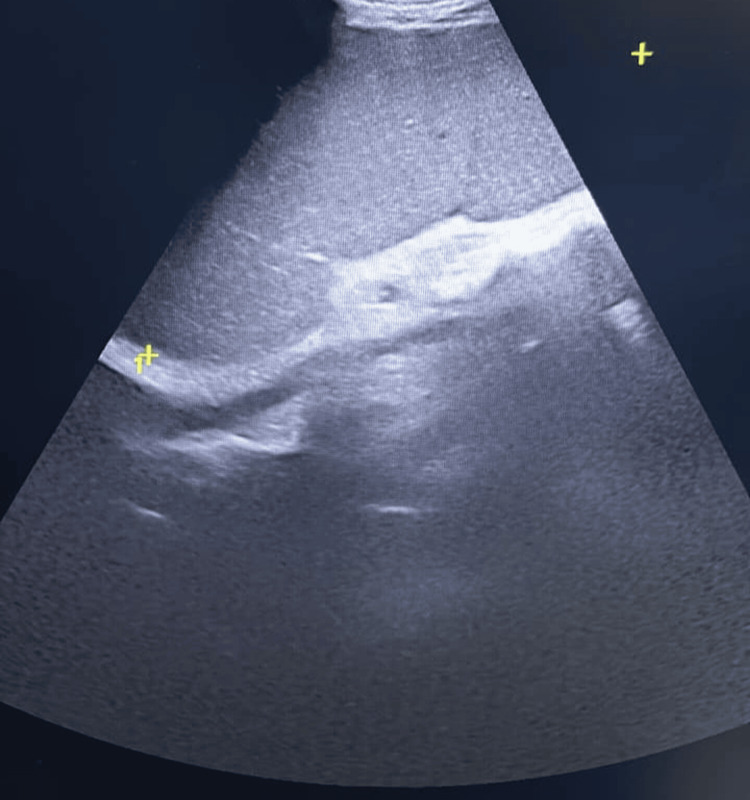
Ultrasonography of the abdomen showing marked splenomegaly.

**Figure 2:**
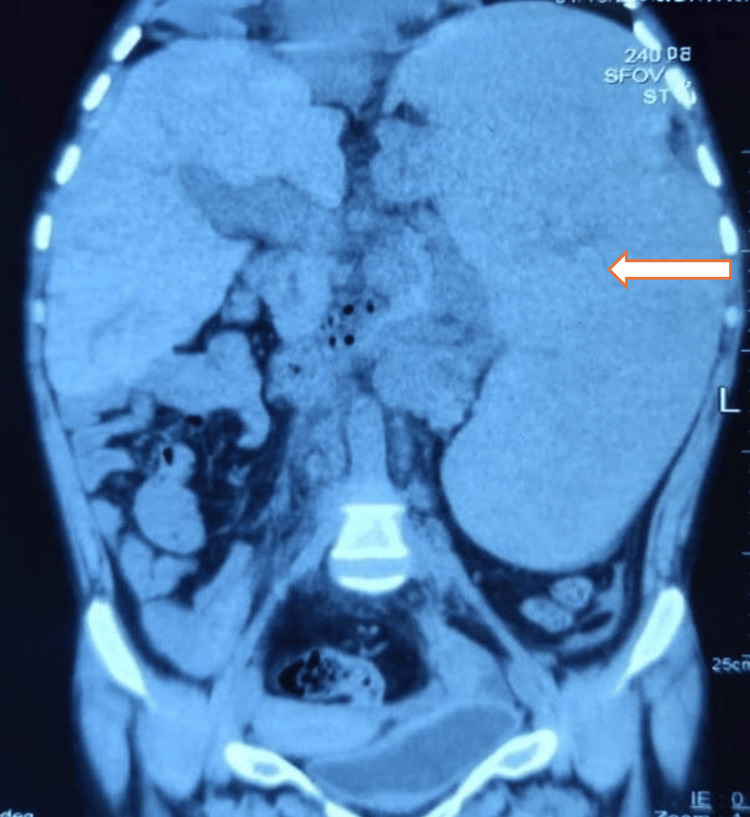
Contrast-enhanced CT of the abdomen (coronal section) showing massive splenomegaly with inferior extension of the spleen (arrow).

Upper gastrointestinal endoscopy revealed large esophageal varices (grade III/IV) with red color signs, indicating a high risk of bleeding. No gastric varices or portal hypertensive gastropathy were identified. Endoscopic variceal band ligation was successfully performed (Figures 3-4), as shown in Figure 3 (pre-banding) and Figure 4 (post-banding).

**Figure 3:**
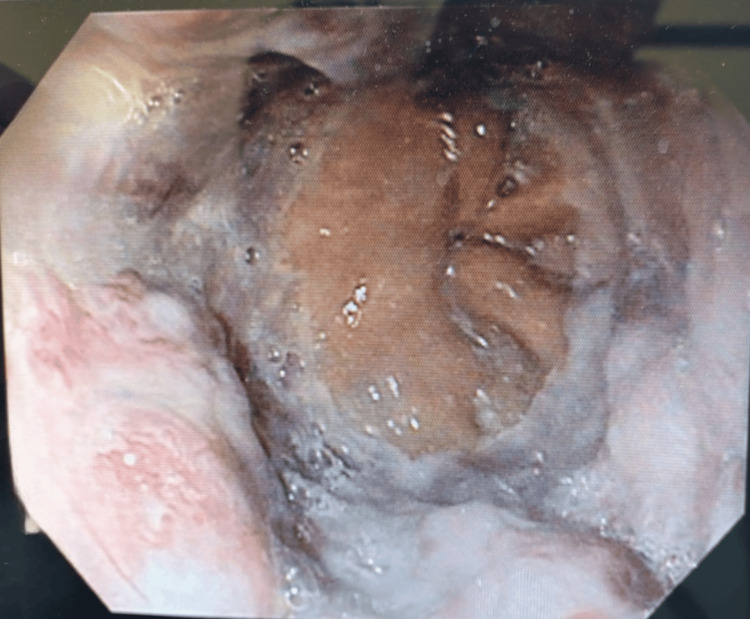
Upper gastrointestinal endoscopy showing large esophageal varices (grade III/IV) with red color signs (RCS) prior to intervention.

**Figure 4:**
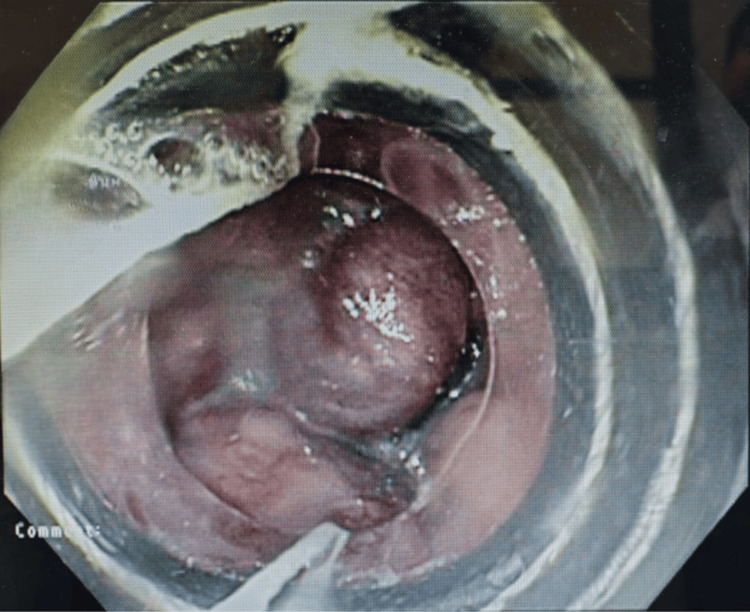
Post-endoscopic band ligation view demonstrating successfully ligated esophageal varices.

Microscopic examination of the bone marrow aspirate revealed increased mast cells with atypical morphology, including spindle-shaped forms and cells with abundant granular cytoplasm. These mast cells were seen in clusters and showed irregular nuclei, consistent with neoplastic mast cell proliferation, as seen in Figure 5. The presence of such atypical mast cells, in conjunction with immunohistochemical positivity for CD117 and CD25, fulfills the World Health Organization (WHO) diagnostic criteria for systemic mastocytosis, with one major criterion in the form of multifocal aggregates of mast cells in the bone marrow, and minor criteria including spindle-shaped atypical mast cells, elevated serum tryptase level, and aberrant CD25 positivity [3]. Although KIT mutation analysis and flow cytometry could not be performed because of financial and logistical limitations, limiting further prognostic stratification and assessment for targeted therapy, the clinicopathological findings were strongly supportive of systemic mastocytosis.

**Figure 5:**
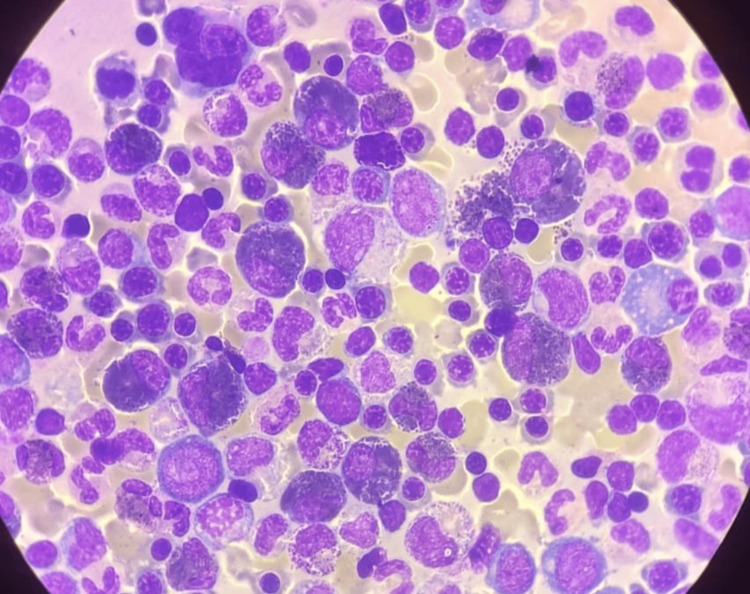
Bone marrow aspirate smear showing numerous atypical mast cells with abundant cytoplasm and dense metachromatic granules. Many cells exhibit spindle-shaped morphology and nuclear irregularity, suggestive of neoplastic mast cell proliferation.

The presence of significant cytopenia, massive hepatosplenomegaly, portal hypertension, and gastrointestinal bleeding suggested an aggressive clinical presentation of systemic mastocytosis rather than an indolent subtype. Based on the clinical, radiological, and histopathological findings, a diagnosis of systemic mastocytosis presenting with non-cirrhotic portal hypertension and upper gastrointestinal bleeding was established. The portal hypertension was likely secondary to mast cell infiltration of the hepatic sinusoids, causing hepatic fibrosis, sinusoidal obstruction, increased intrahepatic vascular resistance, and consequent portal venous hypertension. The patient was managed conservatively with packed red blood cell transfusions and endoscopic variceal band ligation for control of bleeding. She remained hemodynamically stable following the procedure, with no further episodes of gastrointestinal bleeding during hospitalization. In view of the diagnosis of advanced systemic mastocytosis, further evaluation and targeted therapy with tyrosine kinase inhibitors such as midostaurin were advised, as these agents may provide clinical benefit in advanced disease. However, due to financial constraints, targeted therapy could not be initiated. Unfortunately, the patient was subsequently lost to follow-up, limiting long-term assessment of disease progression and treatment outcome.

## Discussion

Systemic mastocytosis is a rare clonal hematological disease characterized by abnormal proliferation and accumulation of mast cells in multiple body organs, predominantly the bone marrow, liver, spleen, and gastrointestinal tract. The disease most often occurs due to activating mutations in the KIT proto-oncogene, most commonly D816V, which result in constitutive activation of mast cells. The clinical manifestations of systemic mastocytosis are diverse, ranging from indolent disease with few symptoms to aggressive manifestations with severe organ dysfunction [1,2]. Variceal bleeding is a rare presentation of systemic mastocytosis, with only a few similar cases reported in the literature [6-8]. In contrast to previously reported cases, which mainly involved older individuals, our patient was a young adult with significant portal hypertension at presentation. The presence of severe cytopenias, massive splenomegaly, portal hypertension, and variceal bleeding suggested an aggressive clinical presentation with significant organ infiltration.

Hepatic involvement in systemic mastocytosis is uncommon but represents an important manifestation of progressive disease. Non-cirrhotic portal hypertension may develop secondary to mast cell infiltration of the hepatic sinusoids and leads to increased intrahepatic vascular resistance [4,5,9]. Unlike cirrhosis, the liver architecture may remain relatively intact, and biochemical abnormalities are usually mild, with a disproportionately elevated alkaline phosphatase level, as seen in our patient. This pathway can be overlooked easily, resulting in delays in diagnosis [6].

The differential diagnosis of non-cirrhotic portal hypertension with splenomegaly and cytopenias includes myeloproliferative neoplasms, lymphomas, infiltrative diseases such as sarcoidosis, and chronic infections. However, abnormal mast cells were identified on bone marrow examination using immunophenotyping, which helped establish the diagnosis of systemic mastocytosis in this case.

In systemic mastocytosis, portal hypertension can be clinically manifested as splenomegaly, hypersplenism, and variceal formation [4,5]. The presence of a massively enlarged spleen in our case also contributes to pancytopenia due to sequestration. However, there are very rare cases presenting with upper gastrointestinal bleeding as a result of varices, which have been reported only in isolated case reports [6-8]. The lack of classic risk factors for chronic liver disease, as well as imaging evidence indicating possible portal hypertension but no cirrhosis, should prompt consideration of other etiologies, such as infiltrative and hematological disorders.

Another important point in this case is the young age at presentation. In younger individuals, systemic mastocytosis is usually indolent and is often characterized by cutaneous manifestations or mediator-related symptoms such as flushing and pruritus [2]. The later development of portal hypertension and extensive organ involvement in a 21-year-old woman is highly uncharacteristic and contributes to the limited literature on aggressive manifestations in young patients.

Diagnosis is still based on bone marrow examination, where typical findings include the presence of dense aggregates of mast cells, spindle-shaped morphology, and aberrant expression of CD25 and CD117 [2,3]. These findings fulfilled the WHO criteria for systemic mastocytosis in our patient. These histopathological features should be considered at an earlier stage, especially when a patient presents with unexplained cytopenias and organomegaly.

Systemic mastocytosis is managed based on the severity and extent of organ involvement. In later stages, specific treatments such as tyrosine kinase inhibitors (e.g., midostaurin) and cytoreductive therapy may be required. Supportive therapy includes antihistamines for mediator-related symptoms and endoscopic treatment of variceal bleeding. Patients with advanced systemic mastocytosis and hepatic involvement generally have a poorer prognosis due to significant organ infiltration and related complications. Early diagnosis is a vital step in ensuring appropriate treatment and preventing complications.

The fact that our patient also has hypothyroidism may indicate an associated autoimmune disorder, but not a direct manifestation of systemic mastocytosis. The role of mast cells in immune regulation has been considered, with their involvement in various inflammatory and autoimmune diseases. Nonetheless, there was no clear causal association between systemic mastocytosis and thyroid dysfunction. Hypothyroidism in this case may thus be incidental or reflect a predisposition toward immune dysregulation.

There are a few important clinical lessons to be noted in this case. First, systemic mastocytosis must be included in the differential diagnosis of non-cirrhotic portal hypertension, particularly in the presence of unexplained cytopenias and massive splenomegaly [6,7]. Second, unusual manifestations may arise in young patients, and a high index of suspicion is essential for timely diagnosis. Lastly, bone marrow examination with appropriate immunohistochemistry is essential for diagnosis and management. Cases with hepatic involvement and portal hypertension may also be associated with poorer clinical outcomes due to significant organ infiltration.

The case had certain limitations, including the unavailability of molecular studies, flow cytometry, and liver stiffness assessment. In addition, long-term follow-up could not be obtained because the patient did not return after discharge.

To conclude, this case highlights a rare and aggressive presentation of systemic mastocytosis with non-cirrhotic portal hypertension and upper gastrointestinal bleeding in a young patient, emphasizing the need to consider infiltrative hematological disorders in unexplained portal hypertension. 

## Conclusions

Systemic mastocytosis is a rare but important cause of non-cirrhotic portal hypertension, particularly in patients with unexplained cytopenias and organomegaly. Although the disease is generally indolent in younger individuals, this case demonstrates that aggressive manifestations, including massive splenomegaly and variceal bleeding, can still occur. This highlights the importance of a high index of suspicion and timely bone marrow examination in atypical presentations. Early diagnosis is essential for accurate disease identification, prompt initiation of appropriate therapy, and prevention of serious complications.
